# Contributors to organ damage in childhood lupus: corticosteroid use and disease activity

**DOI:** 10.1093/rheumatology/keae592

**Published:** 2024-10-22

**Authors:** Maria Hanif, Chandni Sarker, Eslam Al-Abadi, Kate Armon, Kathryn Bailey, Marek Bohm, Mary Brennan, Coziana Ciurtin, Janet Gardner-Medwin, Daniel P Hawley, Alison Kinder, Alice Leahy, Gulshan Malik, Zoe McLaren, Elena Moraitis, Ellen Mosley, Athimalaipet V Ramanan, Satyapal Rangaraj, Annie Ratcliffe, Philip Riley, Heather Rostron, Ethan Sen, Michael W Beresford, Eve M D Smith

**Affiliations:** Department of Women’s & Children’s Health, Institute of Life Course and Medical Sciences, University of Liverpool, Liverpool, UK; Department of Women’s & Children’s Health, Institute of Life Course and Medical Sciences, University of Liverpool, Liverpool, UK; Department of Health Data Science, Institute of Population Health, University of Liverpool, Liverpool, UK; Department of Rheumatology, Birmingham Children’s Hospital, Birmingham, UK; Department of Paediatric Rheumatology, Cambridge University Hospitals, Cambridge, UK; Department of Paediatric Rheumatology, Oxford University Hospitals NHS Foundation Trust, Oxford, UK; Department of Paediatric Rheumatology, Leeds Children Hospital, Leeds, UK; Department of Paediatric Rheumatology, Royal Hospital for Sick Children, Edinburgh, UK; Department of Rheumatology, Centre for Adolescent Rheumatology, University College London, London, UK; Department of Child Health, University of Glasgow, Glasgow, UK; Department of Paediatric Rheumatology, Sheffield Children’s Hospital, Sheffield, UK; Department of Paediatrics, Leicester Children’s Hospital, University Hospitals of Leicester NHS trust, Leicester, UK; Department of Paediatric Rheumatology, Southampton General Hospital, Southampton, UK; Department of Paediatric Rheumatology, Royal Aberdeen Children’s Hospital, Aberdeen, UK; Rheumatology Department, Royal Liverpool and Broadgreen University Hospitals NHS Trust, Liverpool, UK; Department of Paediatric Rheumatology, Great Ormond Street Hospital, London, UK; Department of Paediatrics, Bradford Royal Infirmary, Bradford, UK; Department of Paediatric Rheumatology, University Hospitals Bristol NHS Foundation Trust & Bristol Medical School, University of Bristol, Bristol, UK; Department of Paediatric Rheumatology, Nottingham University Hospitals, Nottingham, UK; Department of Paediatrics, Musgrove Park Hospital, Taunton, UK; Department of Paediatric Rheumatology, Royal Manchester Children’s Hospital, Manchester, UK; Department of Paediatric Rheumatology, Leeds Children Hospital, Leeds, UK; Department of Paediatric Rheumatology, Great North Children’s Hospital & Faculty of Medical Sciences, Newcastle University, Newcastle upon Tyne, UK; Department of Women’s & Children’s Health, Institute of Life Course and Medical Sciences, University of Liverpool, Liverpool, UK; Department of Paediatric Rheumatology, Alder Hey Children’s NHS Foundation Trust Hospital, Liverpool, UK; Department of Women’s & Children’s Health, Institute of Life Course and Medical Sciences, University of Liverpool, Liverpool, UK; Department of Paediatric Rheumatology, Alder Hey Children’s NHS Foundation Trust Hospital, Liverpool, UK

**Keywords:** Childhood SLE, damage, treat-to-target, low disease activity, corticosteroids

## Abstract

**Objectives:**

Awareness of paediatric-specific predictors of damage in childhood lupus is needed to inform mitigation measures. The objective of this study was to ascertain how clinical and demographic variables correlate with damage accrual and identify predictors of damage.

**Methods:**

This analysis included UK JSLE Cohort Study participants. Univariable and multivariable Prentice-Williams-Peterson models investigated how demographic and clinical factors influenced the hazards of new damage. Analyses were performed across the entire cohort, in patients with minimal disease activity marked by a time-adjusted average SLEDAI-2K score (AMS) of ≤2, in patients with low activity (AMS of ≤4), patients with moderate-to-high activity (AMS of >4) and patients with no CS use.

**Results:**

Within the entire cohort (*n* = 430), factors associated with damage included: any methylprednisolone [hazard ratio, HR 2.20 (CI 1.33–3.62)], time-adjusted mean Physician’s Global Assessment (PGA) [HR 2.87 (CI 1.48–5.56)] and AMS score [HR 1.13 (CI 1.03–1.24), all *P < *0.05]. Within the low activity subgroup, any methylprednisolone [HR 2.61 (CI 1.04–6.53)] and time-adjusted mean PGA [HR 3.41 (CI 1.52–7.76)] were associated with damage (both *P < *0.05). Within the moderate-to-high activity subgroup, any methylprednisolone [HR 2.29 (CI 1.31–4.00)], time-adjusted mean PGA [HR 2.66, (CI 1.20–5.87)] and AMS score [HR 1.15 (CI 1.03–1.29)] were predictive of damage (all *P < *0.05). Baseline organ damage was predictive of subsequent damage accrual in the minimal disease activity subgroup [HR 1.33 (CI 1.78–8.08)] and the no CSs subgroup [HR 3.64 (CI 1.83–7.24), both *P < *0.005].

**Conclusion:**

Disease activity levels (AMS/PGA) and proxy indicators (methylprednisolone exposure, baseline damage) were found to be key predictors of damage accrual. This highlights the importance of practical strategies, such as treat-to-target, for reducing disease activity and long-term treatment toxicity.

## Graphical Abstract

**Figure:**
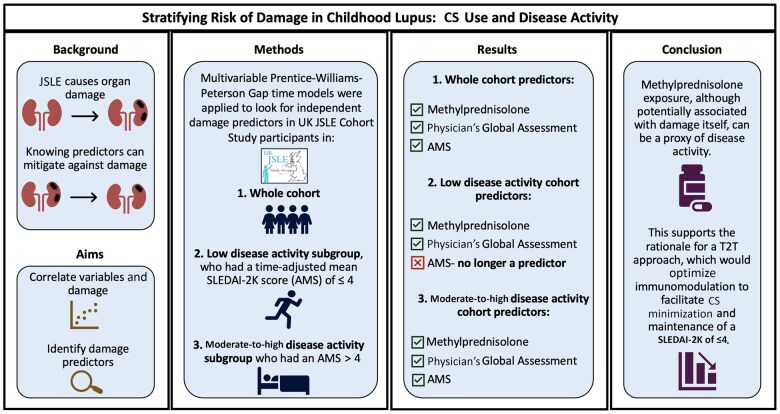

Rheumatology key messages

- Of 430 cSLE patients, 99 (23%) experienced new damage events.
- Potentially modifiable factors associated with damage accrual were methylprednisolone exposure and time-adjusted average SLEDAI-2K score.
- In line with treat-to-target principles, corticosteroid minimization and maintenance of SLEDAI-2K at ≤4 are crucial.

## Introduction

Childhood-onset SLE (cSLE, also known as JSLE) is a chronic autoimmune condition of unknown cause, which causes widespread organ damage due to systemic inflammation [1]. JSLE is a rare disease with an incidence of 0.36–0.46 per 100 000 in the UK [2]. Patients with JSLE generally experience more severe disease than their adult counterparts, with increased levels of renal, haematological and neurological system involvement [3, 4]. In order to reduce disease-related damage, morbidity and associated mortality, as well as improve health-related quality of life, a ‘treat to target’ (T2T) strategy has been proposed for cSLE patients [5].

JSLE treatment consists of traditional DMARDs, biologic immunotherapies and CSs, for example oral prednisolone and/or i.v. methylprednisolone [6]. CS therapy rapidly controls inflammation, enabling immunosuppressive therapies to accumulate to their therapeutic level [7]. Despite the effectiveness of CS therapy for reducing systemic inflammation and controlling JSLE flares [8], these treatments have a multitude of potential adverse effects [9, 10]. Multiple studies have noted the damage caused by CS therapy in both adult and paediatric SLE populations [11–13], with such adverse effects noted to be increased in JSLE patients compared with adult-onset SLE (aSLE) patients [14].

There is limited literature on the longer-term predictors of organ damage in JSLE, and the relative contribution of CS treatment to damage outcomes as compared with clinical and demographic factors. Awareness of predictors of damage that are potentially modifiable is particularly important for the optimization of care, and for the prevention of damage. The aim of this study was to identify independent predictors of damage in JSLE patients according to their disease activity level during follow-up, and to identify predictors that are relevant in patients with differing disease trajectories.

## Methods

### Patient cohort

Longitudinal real-world patient data was obtained from the UK JSLE Cohort Study, a large multicentre study conducted across 22 paediatric rheumatology sites in the UK, spanning 2006 to February 2020 [15]. To be eligible, patients at diagnosis were ≤18 years old and had achieved a  ≥4 ACR classification criteria for SLE [16]. The majority of the patients were recruited at the time of diagnosis or shortly after; however, this study was also open to patients later on in their disease course. Many of the UK JSLE Cohort Study centres faced an interruption in data collection during the pandemic, as UK research shifted focus toward COVID-19. To maintain the integrity of the longitudinal analysis, we used data gathered before the pandemic (prior to February 2020). Prior to any data collection, patient and parental consent was obtained (National Research Ethics Service Northwest, Liverpool, 06/Q1502/77), and all research was undertaken in accordance with the Declaration of Helsinki.

### Clinical predictors of damage

Many factors were investigated to determine whether they influenced the hazards of damage accrual in JSLE patients. These included demographic factors: age, gender, ethnicity (with mixed ethnicity patients being grouped according to their ethnicity minority group); medication factors: any prednisolone exposure, time-adjusted mean prednisolone exposure (averaged over time), any methylprednisolone exposure, any immunosuppressant exposure, any HCQ exposure; clinical factors: time-adjusted mean Physician’s Global Assessment (PGA, averaged over time) [17], baseline organ damage (measured using the SLICC/ACR Damage Index score, SLICC-SDI score) [18], baseline renal activity (measured using the BILAG score) [19], and overall disease activity during follow-up (measured using an adjusted mean SLEDAI-2K score, AMS score) [20]. Baseline is defined as at the time of study entry. Of note, the exact dose of methylprednisolone was not collected by the UK JSLE Cohort Study. Instead, whether a patient had <5 pulses or ≥5 pulses was recorded; therefore, the term any methylprednisolone exposure is used as the variable. Factors were first investigated to determine whether there was any correlation using a heatmap (Supplementary Fig. S1, available at *Rheumatology* online). As the variables ‘any prednisolone exposure’ and ‘time-adjusted mean prednisolone exposure’ were correlated (*r* > 0.5), the variable ‘any prednisolone exposure’ was removed from the analyses.

### Patient groups

Predictors of damage accrual were initially investigated in the whole cohort, and then in four subgroups. Three of these subgroups were disease activity state subgroups, defined according to the AMS value over the course of a patient’s follow-up. These reflected the trajectory of each patient’s disease course. The following disease activity state subgroups were included: (i) moderate-to-high disease activity (AMS > 4); (ii) low disease activity [AMS ≤ 4, in line with the childhood lupus low disease activity state (cLLDAS) disease activity entry criterion] [21]; (iii) minimal disease activity, depicted by an AMS score of ≤2 throughout follow-up. The fourth subgroup involved patients who had no CS therapy (no prednisolone or methylprednisolone) throughout the observed follow-up period, to identify predictors of damage accrual that were independent of CS therapy.

### Outcome definition

The main outcome assessed in this study was damage accrual, defined by a SLICC-SDI score increase of ≥1 point at any time point during the follow-up period [18, 22].

### Statistical analysis

#### Time-adjusted variables

Factors involving time-adjusted measurements, including mean prednisolone exposure, mean PGA score and adjusted mean SLEDAI-2K score (AMS) had their values averaged, considering the varying lengths of follow-up and the timing of the assessments. To calculate the AMS score, a patient’s SLEDAI-2K value at every visit was collected and the average was calculated before being adjusted for the time between visits. This provided a more comprehensive summary view of a patient’s disease activity, rather than relying on single-point measurements. This same principle was applied to all other time-adjusted values [20]. A graphical depiction of AMS calculation is depicted in Supplementary Fig. S2, available at *Rheumatology* online.

#### Descriptive analyses of clinical and demographic characteristics

Descriptive analyses were conducted to assess baseline clinical and demographic characteristics, and were summarized using medians and interquartile ranges, or frequencies and percentages, for continuous and categorical variables, respectively. To determine whether there were any significant differences between characteristics of the low disease activity subgroup and the moderate-to-high disease activity subgroup, Chi-squared and Mann–Whitney U tests were conducted for categorical and continuous variables, respectively.

#### Exploration of the impact of predictors on damage accrual

The main outcome assessed in this study was damage accrual, and as this was considered a recurrent event, which required every visit to be taken into account, univariable Prentice-Williams-Peterson (PWP) Gap time models were implemented to assess the impact of several factors individually on damage accrual [23], using the ‘Survival’ R package [‘coxph()’ R function] [24]. Factors that were found to be significant from univariable analyses (*P < *0.05) were then adjusted for in multivariable PWP models using the ‘MASS’ R package via backwards selection [‘stepAIC()’ R function] [25] to determine whether any of the factors were independent damage predictors. Results were presented as hazard ratios (HRs) with 95% CIs, with statistically significant factors having a *P*-value of <0.05. These analyses were undertaken in the whole cohort, and in the four subgroups of patients. Multivariable analysis was not conducted on the minimal disease activity subgroup, as only one factor was significant on univariable analysis. All analyses were conducted using R (Version 4.3.2) [26].

## Results

### Patients

**Figure 1.**
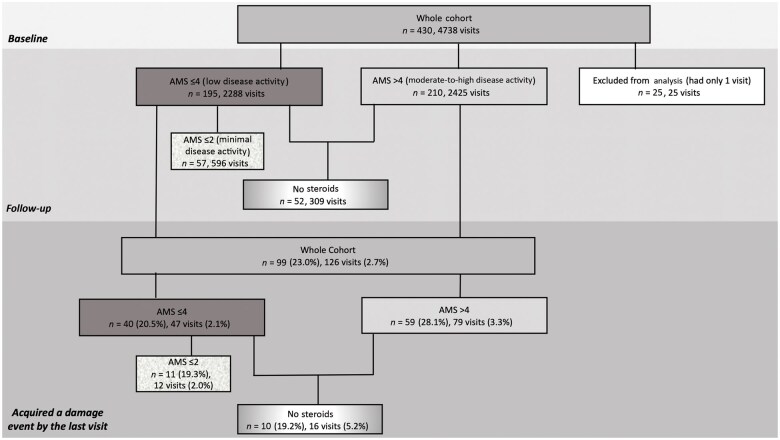
Flow chart depiction of patients and visits with damage. Excluded patients only had a single study visit (AMS could not be calculated). AMS: Adjusted Mean SLEDAI-2K

The whole cohort consisted of 430 JSLE patients, with a total of 4738 visits recorded. Clinical and demographic characteristics of the whole cohort, each of the disease-activity subgroups, and those who did not receive CSs during the observed follow-up period are summarized in Table 1. The median follow-up time was 46 months (IQR: 18–63) with a median of 10 visits per patient (IQR: 5–15). The cohort constituted of a majority of females (83.5%), with half of the cohort being of White ethnicity (52.1%). The median age at diagnosis was 12.8 years (IQR: 10.4–14.6). At baseline, 16.1% of patients had organ damage. During subgroup analysis, 25 patients were excluded, as they only had one visit, see Fig. 1. Statistical differences in clinical and demographic factors at baseline between the low disease activity and moderate-to-high disease activity subgroups are shown in Supplementary Table S1, available at *Rheumatology* online.

**Table 1.** Clinical and demographic features

Clinical/Demographic factors	Description	All patients (*n* = 430)	Sub-groups of patients			
			Adjusted mean SLEDAI-2K ≤4 patients (*n* = 195, low disease activity)	Adjusted mean SLEDAI-2K >4 patients (*n* = 210)	Moderate-to-high disease activity) adjusted mean SLEDAI-2K ≤2 patients (*n* = 57)	Minimal disease activity) no CSs (*n* = 52)
**Median number of visits (IQR)**	Per patient	10 (5–15)	10 (6–16)	10 (5–15)	9 (5–15)	4 (2–8)
**Median time between first ever visit and diagnosis date (IQR)**	In months	0.0 (0.0–2.0)	0.0 (0.0–3.3)	0.0 (0.0–0.9)	0.0 (0.0–4.6)	0.0 (0.0–4.9)
**Median disease duration (IQR)**	In years	3.6 (2.0–5.8)	4.1 (2.5–6.5)	3.6 (1.9–5.6)	4.1 (2.4–5.9)	2.5 (1.0–4.5)
**Median time interval between visits (IQR)**	In months	3.2 (2.1–4.8)	3.5 (2.3–5.4)	3.0 (1.8–4.4)	3.5 (2.4–5.3)	3.5 (2.3–5.8)
**Gender, *n* (%)**	Female	359 (83.5)	162 (83.1)	176 (83.8)	41 (71.9)	43 (82.7)
**Median age (IQR)**	At diagnosis	12.8 (10.4–14.6)	12.5 (10.1–13.9)^c^	12.9 (10.6–14.8)^c^	11.6 (9.6–13.9)	13.2 (12.6–15.9)
**Ethnicity, *n* (%)** ^a^	African/Caribbean	72 (17.2)	27 (14.7)	37 (18.8)	8 (14.8)	6 (12.2)
	Asian	129 (30.8)	51 (27.7)	64 (32.5)	20 (37.0)	14 (28.6)
	White	218 (52.0)	106 (57.6)	96 (48.7)	26 (48.2)	29 (59.2)
**Baseline organ damage SDI score, *n* (%)** ^b^	No damage 0	344 (84.0)	160 (86.0)	166 (82.2)	46 (86.8)	44 (89.9)
	Mild damage (1)	49 (12.0)	21 (11.3)	25 (12.4)	5 (9.4)	3 (6.1)
	Moderate damage (2)	9 (2.0)	2 (1.1)	6 (3.0)	1 (1.9)	1 (2.0)
	Severe damage (≥3)	8 (2.0)	3 (1.6)	5 (2.4)	1 (1.9)	1 (2.0)
**New damage accrued during follow up period, *n* (%)**	No damage 0	0 (0)	0 (0)	0 (0)	0 (0)	0 0
	Mild damage (1)	54 (54.5)	22 (55.0)	32 (54.2)	7 (63.6)	7 (70.0)
	Moderate damage (2)	20 (20.2)	9 (22.5)	11 (18.6)	3 (27.3)	2 (20.0)
	Severe damage (≥3)	25 (25.3)	9 (22.5)	16 (27.1)	1 (9.1)	1 (10.0)
**Median ACR score at last visit (IQR)**	At baseline	5 (5–7)	5 (4–6)^c^	6 (5–7)^c^	5 (4–6)	5 (4–5)
**Any steroids during follow-up, *n* (%)**	Yes	378 (87.9)	169 (86.7)	195 (92.9)	45 (78.9)	NA
**Prednisolone during follow-up, *n* (%)**	Yes	371 (86.3)	166 (85.1)	192 (91.4)	43 (75.4)	NA
**Time-adjusted mean prednisolone (IQR)**	During follow-up	5.5 (1.9–9.8)	3.8 (1.1–7.4)^c^	7.6 (4.0–11.9)^c^	2.9 (0.1–5.6)	NA
**Methylprednisolone during follow-up, *n* (%)**	Yes	201 (46.7)	76 (39.0)^c^	119 (56.7)^c^	19 (33.3)	NA
**Number of methylprednisolone pulses**	No pulses ever	229 (53.3)	119 (61.0)	91 (43.3)	38 (66.7)	NA
	<5 pulses ever	141 (32.8)	53 (27.2)	83 (39.5)	14 (24.6)	
	≥5 pulses ever	60 (14.0)	23 (11.8)	36 (17.1)	5 (8.8)	
**HCQ during follow-up, *n* (%)**	Yes	394 (91.6)	185 (94.9)	192 (91.4)	53 (93.0)	45 (86.5)
**Immunosuppressants during follow-up, *n* (%)**	Yes	423 (98.4)	195 (100.0)	208 (99.0)	57 (100)	47 (90.4)
**Baseline renal BILAG disease activity score, *n* (%)**	Severe-to-moderately active disease	142 (33.0)	62 (31.8)	71 (33.8)	15 (26.3)	4 (7.7)
**Time-adjusted mean PGA score (IQR)**	During follow-up	0.13 (0.03–0.32)	0.08 (0.02–0.23)^c^	0.17 (0.05–0.40)^c^	0.05 (0.01–0.15)	0.06 (0.00–0.26)

Data presented as frequencies/counts or medians/interquartile ranges (IQRs). Of note, the number of patients in the four subgroups does not add up to *n* = 430 due to patients featuring in multiple subgroups.

a Ethnicity data was unavailable for 11 patients.

b SDI data was unavailable for 20 patients. There were 25 patients were excluded from Adjusted Mean SLEDAI-2K (AMS) analysis, as they had one visit. Immunosuppressants included: AZA, MMF, Cyclosporine A, MTX, IVIG G, rituximab and CYC.

c Significant difference (*P < *0.05) demonstrated when comparing these factors between those with low disease activity and moderate-to-high disease activity (Supplementary Table S1, available at *Rheumatology* online). NA: not applicable (patients not on CSs); ACR: American College of Rheumatology Classification Criteria, PGA: Physician’s Global Assessment; SDI: SLICC/ACR Damage Index.

### Damage events

**Figure 2.**
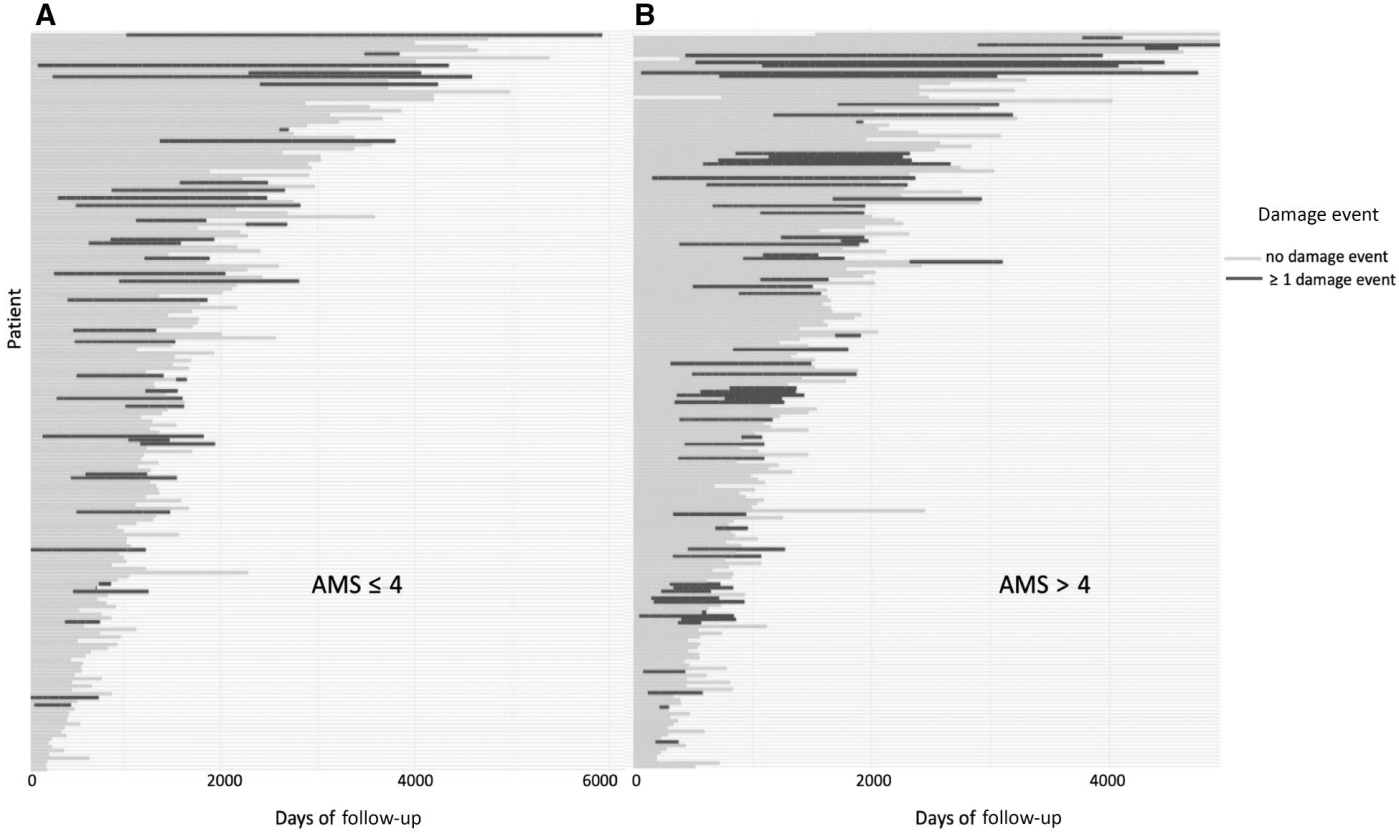
Graphical representation of damage events over the course of follow-up, patients who have been stratified according to Adjusted Mean SLEDAI-2K (AMS) score. Damage event is defined as the time point of first new damage development. (A) Damage events for patients with low disease activity (AMS ≤ 4) throughout the follow-up period. (B) Damage events for patients with moderate-to-high disease activity (AMS > 4) throughout the follow-up period. AMS: Adjusted Mean SLEDAI-2K

**Figure 3.**
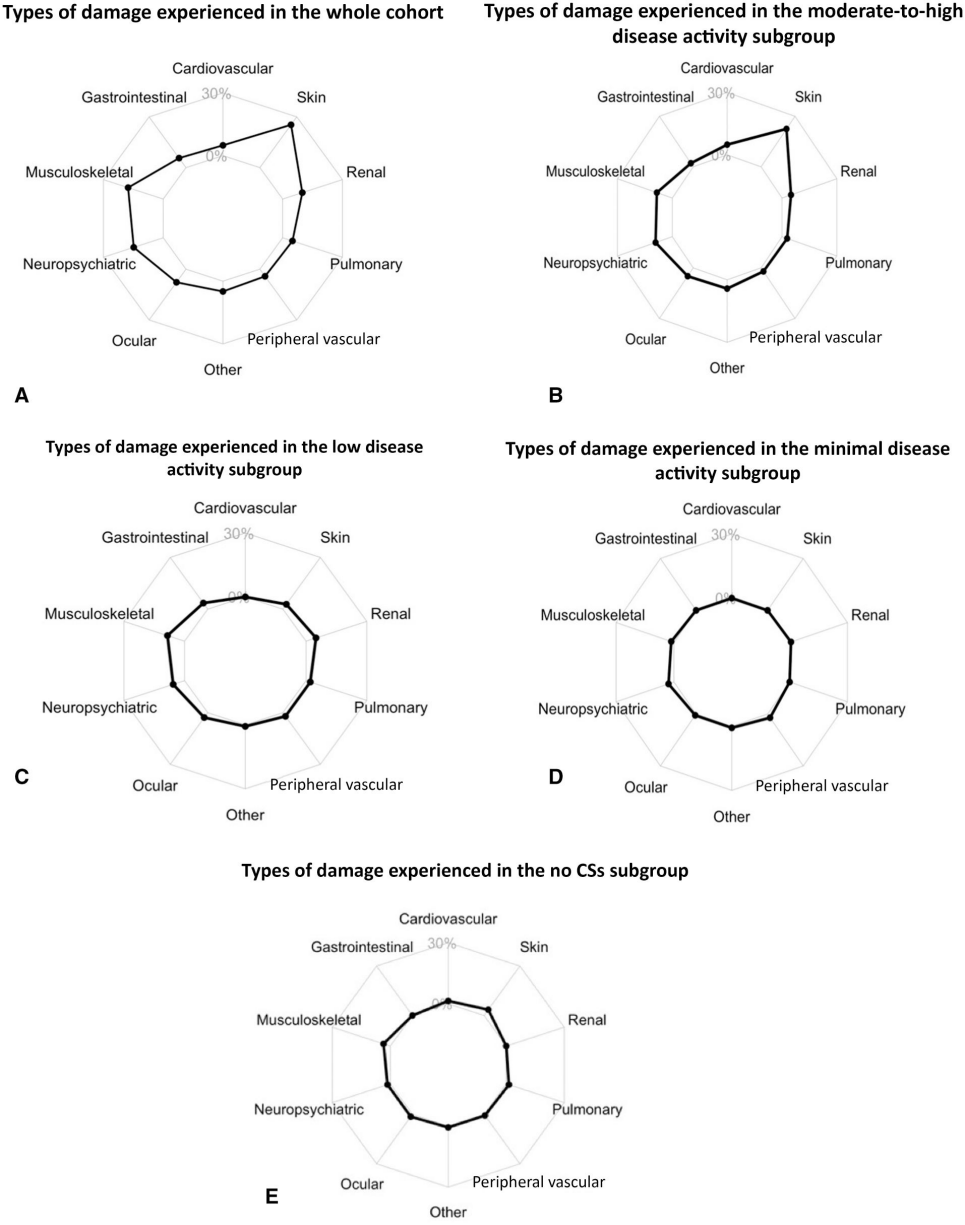
Radar charts demonstrating the types of damage accrued. (A) Whole cohort damage types. (B) Moderate-to-high disease activity subgroup damage types. (C) Low disease activity subgroup damage types. (D) Minimal disease activity subgroup damage types. (E) No CSs subgroup damage types. Damage included: cardiovascular: pericarditis, valvular disease, myocardial infarction, angina, cardiomyopathy; skin: alopecia, panniculus, ulceration; renal: end-stage renal disease, eGFR of <50%, proteinuria; pulmonary: hypertension, fibrosis, shrinking lung, infarction; peripheral vascular; tissue loss, venous thrombosis; ocular; retinal change, cataract; neuropsychiatric; cognitive impairment, seizures, cerebral vascular accident, neuropathy, transverse myelitis; musculoskeletal: arthritis, atrophy, osteoporosis, avascular necrosis, osteomyelitis, tendon rupture; gastrointestinal; infarction, mesenteric insufficiency, peritonitis, stricture, pancreatic insufficiency; other: diabetes, malignancy, gonadal failure

Within the whole cohort, of the 410 patients for whom this data was available, 66 patients had organ damage at baseline (16.0%). From the start to the end of the follow-up period, a further 99/430 patients (23.0%) experienced new organ damage. Seventeen patients demonstrated both baseline organ damage and new organ damage during follow-up. Therefore, overall, by the last follow-up visit, 148/430 (34.4%) patients had experienced some form of organ damage (see Supplementary Fig. S3, available at *Rheumatology* online). The moderate-to-high disease activity subgroup (AMS > 4) had the highest proportion of patients experiencing new damage events during follow-up [59/210 (28.1%)], while the lowest proportion was in the no CS subgroup [10/52 (19.2%)] (see Table 1). Fig. 1 summarizes the total number of visits and patients who experienced new damage, both for the whole cohort and for each subgroup of patients. Fig. 2 shows the temporal occurrence of new damage events during follow-up in patients with low disease activity (AMS ≤ 4) and moderate-to-high disease activity (AMS > 4), demonstrating that more new damage events took place in the moderate-to-high disease activity subgroup. The types of damage experienced by the whole cohort are described in Fig. 3A. Fig. 3B–E shows the types of damage accrued in each patient disease activity subgroup (moderate-to-high, low, minimal disease activity, and no CS). Skin-related damage was commonest across the whole cohort, in particular within those with moderate-to-high disease activity (Fig. 3A and B).

### Predictors of damage across the whole cohort

Within the whole cohort, factors significantly associated with damage accrual in univariable analyses included: time-adjusted mean prednisolone dose [HR 1.05 (CI 1.01–1.09), *P = *0.007]; time-adjusted mean PGA score [HR 5.08 (CI 2.82–9.16), *P < *0.001]; any immunosuppressant exposure [HR 2.51 (CI 1.02–6.21), *P = *0.045]; any methylprednisolone exposure [HR 3.05 (CI 1.89–4.91), *P < *0.001]; and AMS score [HR 1.17 (CI 1.10–1.25), *P < *0.001] (Table 2). For the following factors: time-adjusted prednisolone dose, time-adjusted PGA score and AMS score, a 1-unit increase in the score, or dosage was associated with the reported increase in the unadjusted hazards of damage. Within the multivariable model, three independent predictors of damage remained, namely: any methylprednisolone exposure [HR 2.20 (CI 1.33–3.62), *P = *0.002]; time-adjusted mean PGA score [HR 2.87 (CI 1.48–5.56), *P = *0.002]; and AMS score [HR 1.13 (CI 1.03–1.24), *P = *0.013] (see Table 3).

**Table 2.** Univariable PWP gap-time models exploring predictors of new damage accrual in the whole cohort and subgroups of patients

Factor	Whole cohort	AMS ≤4 Patients (*n* = 195, low disease activity)	AMS >4 Patients (*n* = 210, moderate-to-high disease activity)	AMS ≤2 Patients (*n* = 57, minimal disease activity)	No CSs patients (*n* = 52)
**Age at diagnosis **	HR 1.04, CI (0.96–1.12) *P = *0.372	HR 1.05, CI (0.97–1.15) *P = *0.209	HR 1.02, CI (0.98–1.16) *P = *0.814	HR 1.01, CI (0.88–1.16) *P = *0.874	HR 0.84, CI (0.57–1.23) *P = *0.371
**Ethnicity**					
** Black Caribbean compared with White**	HR 0.74, CI (0.44–1.25) *P = *0.259	HR 0.62, CI (0.23–1.65) *P = *0.342	HR 0.91, CI (0.50–1.65) *P = *0.747	HR 1.29, CI (0.89–8.87) *P = *0.799	HR 2.38, CI (0.54–10.45) *P = *0.251
** Black Caribbean compared with Asian**	HR 0.91, CI (0.53–1.56) *P = *0.732	HR 0.99, CI (0.36–2.68) *P = *0.978	HR 0.93, CI (0.49–1.75) *P = *0.821	HR 2.97, CI (0.58–15.21) *P = *0.191	HR 0.62, CI (0.06–6.31) *P = *0.687
** Asian compared with White British**	HR 0.82, CI (0.52–1.28) *P = *0.376	HR 0.63, CI (0.34–1.18) *P = *0.148	HR 0.98, CI (0.53–1.79) *P = *0.936	HR 0.43, CI (0.12–1.59) *P = *0.208	HR 3.83, CI (0.49–29.66) *P = *0.198
**Time-adjusted mean prednisolone dose**	**HR 1.05, CI (1.01–1.09)** ***P = *0.007**	HR 1.05, CI (0.98–1.12) *P = *0.188	HR 1.03, CI (0.98–1.08) *P = *0.202	HR 0.91, CI (0.74–1.12) *P = *0.373	NA
**Disease duration**	HR 0.95, CI (0.83–1.09) *P = *0.456	HR 0.91, CI (0.72–1.14) *P = *0.413	HR 1.01, CI (0.85–1.19) *P = *0.942	HR 1.31, CI (0.94–1.84) *P = *0.111	HR 0.94 (0.63–1.42) *P = *0.771
**Gender**	HR 1.18, CI (0.70–2.00) *P = *0.532	HR 1.80, CI (0.70–4.64) *P = *0.221	HR 1.07, CI (0.57–2.02) *P = *0.827	HR 3.06, CI (0.41–23.00) *P = *0.277	^a^
**Time-adjusted mean PGA score **	**HR 5.08, CI (2.82–9.16)** ***P < *0.001**	**HR 3.53, CI (1.57–7.91)** ***P = *0.002**	**HR 4.19, CI (1.92–9.17)** ***P < *0.001**	HR 6.04, CI (0.82–44.6) *P = *0.078	**HR 34.15, CI (1.06–1100)** ***P = *0.046**
**Any immunosuppressants **	**HR 2.51, CI (1.02–6.21)** ***P = *0.045**	HR 1.75, CI (0.58–5.30) *P = *0.322	HR 3.65, CI (0.84–15.88) *P = *0.084	HR 2.20, CI (0.24–19.82) *P = *0.481	HR 0.70, CI (0.20–2.51) *P = *0.589
**Any methylprednisolone**	**HR 3.05, CI (1.89–4.91)** ***P < *0.001**	**HR 2.72, CI (1.12–6.60)** ***P = *0.027**	**HR 2.76, CI (1.57–4.87)** ***P < *0.001**	^a^	NA
**Any HCQ**	HR 1.30, CI (0.84–2.03) *P = *0.243	HR 1.35, CI (0.68–2.66) *P = *0.389	HR 1.30, CI (0.76–2.23) *P = *0.346	HR 1.72, CI (0.40–7.39) *P = *0.469	HR 0.53, CI (0.16–1.72) *P = *0.287
**AMS score**	**HR 1.17, CI (1.10–1.25)** ***P < *0.001**	HR 0.96, CI (0.70–1.33) *P = *0.828	**HR 1.78, CI (1.09–1.28)** ***P < *0.001**	HR 0.98, CI (0.37–2.60) *P = *0.975	**HR 1.25, CI (1.10–1.42)** ***P < *0.001**
**Baseline organ damage**	HR 1.25, CI (0.91–1.72) *P = *0.162	HR 1.37, CI (0.94–2.00) *P = *0.100	HR 1.17, CI (0.79–1.73) *P = *0.425	**HR 1.33, CI (1.78–8.08)** ***P = *0.001**	**HR 3.71, CI (1.79–7.68)** ***P < *0.001**
**Baseline Renal Disease**	HR 1.09, CI (0.73–1.65) *P = *0.667	HR 1.17, CI (0.61–2.26) *P = *0.633	HR 1.09, CI (0.66–1.82) *P = *0.737	HR 0.72, CI (0.16–3.26) *P = * 0.675	^a^

a Number of events for these factors were too low so a model could not be fitted. Immunosuppressants included: AZA, MMF, Cyclosporin A, MTX, IVIG G, rituximab and CYC. NA: not applicable as these patients were not on CSs; AMS: Adjusted Mean SLEDAI-2K; PGA: Physician’s Global Assessment; PWP: Prentice-Williams-Peterson.

**Table 3.** Multivariable PWP gap-time models exploring predictors of new damage accrual in the whole cohort and subgroups of patients

Model	Factors	Hazard ratio (95% CI)	*P*-value
**Whole cohort *n* = 430**	**Time-adjusted mean PGA score**	**HR 2.87 (CI 1.48–5.56)**	***P = *0.002**
	**Any methylprednisolone**	**HR 2.20 (CI 1.33–3.62)**	***P = *0.002**
	**AMS**	**HR 1.13 (CI 1.03–1.24)**	***P = *0.013**
**AMS ≤4 patients *n* = 195 (low disease activity)**	**Time-adjusted mean PGA score**	**HR 3.41 (CI 1.52–7.67)**	***P = *0.003**
	**Any methylprednisolone**	**HR 2.61 (CI 1.04–6.53)**	***P = *0.040**
**AMS >4 patients *n* = 210 (moderate-to-high disease activity)**	**Time-adjusted mean PGA score**	**HR 2.66 (CI 1.20–5.87)**	***P = *0.016**
	**Any methylprednisolone**	**HR 2.29 (CI 1.31–4.00)**	***P = *0.004**
	**AMS**	**HR 1.15 (CI 1.03–1.29)**	***P = *0.014**
**No CSs patients *n* = 52**	**Baseline organ damage**	**HR 3.64 (CI 1.83–7.24)**	***P < *0.001**

AMS: Adjusted Mean SLEDAI-2K; PGA: Physician’s Global Assessment; PWP: Prentice-Williams-Peterson.

### Predictors of damage for those with moderate-to-high disease activity during follow-up

Within the moderate-to-high disease activity subgroup (AMS > 4 during follow-up), the factors significantly associated with damage accrual in univariable analyses were: time-adjusted mean PGA score [HR 4.19 (CI 1.92–9.17), *P < *0.001]; any methylprednisolone exposure [HR 2.76 (CI 1.57–4.87), *P < *0.001]; and AMS score [HR 1.78 (CI 1.09–1.28), *P < *0.001] (Table 2). All these factors remained significant independent predictors of damage when applied to the multivariable model, namely: any methylprednisolone exposure [HR 2.29 (CI 1.31–4.00), *P = *0.004]; time-adjusted mean PGA score [HR 2.66 (CI 1.20–5.87), *P = *0.016]; and AMS score [HR 1.15 (CI 1.03–1.29), *P = *0.014] (Table 3).

### Damage predictors for those in low disease activity during follow-up

Within the low disease activity subgroup (AMS ≤ 4 during follow-up), only two factors were associated with damage accrual within the univariable analyses, namely: time-adjusted mean PGA score [HR 3.53 (CI 1.57–7.91), *P = *0.002] and any methylprednisolone exposure [HR 2.72 (CI 1.12–6.60), *P = *0.027] (Table 2). Both factors remained as significant predictors of damage in the multivariable model: methylprednisolone exposure [HR 2.61 (CI 1.04–6.53), *P = *0.040]; and time-adjusted mean PGA score [HR 3.41 (CI 1.52–7.76), *P = *0.003] (Table 3).

### Damage predictors for those with minimal disease activity and no CS treatment during follow-up

Within the minimal disease activity subgroup (AMS score of ≤2 throughout follow-up), only baseline organ damage (baseline SDI score) was a significant predictor of damage accrual [HR 1.33 (CI 1.78–8.08), *P = *0.001]. A multivariable model could not, therefore, be created (Table 2). With regards to the subgroup of patients who did not receive any CSs during the observed follow-up period, in the univariable analyses, time-adjusted mean PGA score [HR 34.15 CI (1.06–1100), *P = *0.046]; AMS score [HR 1.25 (CI 1.10–1.42), *P < *0.001]; and baseline organ damage [HR 3.71 (CI 1.79–7.68), *P < *0.001] were associated with damage accrual (Table 2). When these factors were applied to a multivariable model, only baseline organ damage remained as a significant independent predictor of damage [HR 3.64 (CI 1.83–7.24), *P < *0.001] (Table 3).

## Discussion

Damage in JSLE is associated with an increase in morbidity, mortality, and poor health-related quality of life [1, 5]. Within the current study, 34.4% of the whole cohort had experienced damage accrual by the end of the follow-up period, with 23.0% of the whole cohort experiencing new damage events (additional to any pre-existing baseline damage). There was a higher proportion of damage accrued in the moderate-to-high disease activity subgroup (28.1% of patients), and a lower proportion of damage accrued in the low disease activity subgroup (20.5%). This is in keeping with the findings of previous studies assessing the impact of LLDAS attainment on damage accrual [27]. To minimize damage accrual, it is necessary to be aware of potentially modifiable predictors of damage. Across the patient subgroups, the potentially modifiable factors that were most consistently associated with increased risk of damage accrual included exposure to i.v. methylprednisolone during follow-up, and the time-adjusted mean AMS and PGA scores. I.v. methylprednisolone exposure, although potentially associated with damage itself, can also be regarded as a proxy for disease activity. Both the AMS and PGA scores also reflected fluctuations in disease activity over the follow-up period. These data support the rationale for a T2T approach, whereby pursuit of targets such as cLLDAS would promote optimization of immunomodulation to facilitate CS minimization, and attainment and maintenance of a SLEDAI-2K score of ≤4 during follow-up.

The type of damage experienced most within the whole cohort was mucocutaneous-related (ulcers, alopecia, and panniculus). Skin-related damage is disturbing to young patients, serving as a physical reminder of their disease, and is also easy to observe in patients during examination, as compared with other forms of damage that require investigations in order to be identified [28]. Musculoskeletal damage (e.g. osteoporosis and avascular necrosis) was the most prevalent damage type for those with low disease activity, and is often related to CS treatment [28, 29]. Though skin damage may appear to have been high, this was in line with the findings of another study, which showed a similar proportion of skin damage accrued at the approximate same time of follow-up to this study [30].

The multivariable models from the whole cohort, and the moderate-to-high disease subgroup, both demonstrated that methylprednisolone exposure, PGA score and AMS score were associated with damage accrual. Conversely, within the low disease activity subgroup multivariable model, the AMS score was not statistically significantly associated, suggesting that increases in the SLEDAI-2K score up to a maximum of 4 points does not impact significantly on damage accrual. These observations support the results of previous studies, which have indicated that maintenance of a LLDAS state (SLEDAI-2K of ≤4 during follow-up) protects against damage accrual [11, 27], and that CS exposure is associated with damage accrual in aSLE [11, 12].

Any methylprednisolone exposure and time-adjusted mean prednisolone dose were both significant predictors of damage in the univariable analysis, with any methylprednisolone remaining a significant predictor of damage in the multivariable analyses. Recommendations for the management of JSLE, such as the SHARE recommendations, suggest that when a pulse of methylprednisolone is required, 30 mg/kg, maximum 1 g should be used [31]. This guidance was published in 2017, and since then newer aSLE recommendations (including the EULAR 2023 SLE Guidelines and the KDIGO Lupus Nephritis Guidelines) have advocated for capping the methylprednisolone dose at 250 mg or 500 mg per pulse in most clinical situations [32–35]. In the UK, the British Society of Rheumatology is currently working towards the development of guidelines for the management of SLE across the life-course [36]. It is, therefore, anticipated that these newer guidelines will help to align methylprednisolone dosing between JSLE and aSLE patients.

While methylprednisolone exposure was statistically associated with damage accrual, we cannot conclude that it is a direct cause of damage. Generally, methylprednisolone was followed by prolonged oral prednisolone use in these patients. Therefore, it could be the subsequent prednisolone dose and/or duration of prednisolone exposure that could also have contributed to the observed damage, as the time-adjusted mean prednisolone dose was averaged out in a way that assumed linear tapering. The UK JSLE Cohort Study does not collect detailed information on the specific methylprednisolone regimes used. The impact of different methylprednisolone treatment regimens cannot, therefore, be assessed further in the current study. These data and considerations highlight the need for more research to optimize use of i.v. methylprednisolone regimens to inform clinical practice decision-making, potentially reducing the risk of damage without compromising lupus control [37].

With regards to the minimal disease activity subgroup (AMS score of ≤2 throughout follow-up) and the no CSs subgroup, baseline organ damage was demonstrated to be a predictor of subsequent damage accrual. This observation is in keeping with the results of aSLE studies, which have shown that those who develop damage early in the disease accrue subsequent damage at a faster rate [11, 38]. This finding supports the need to treat patients aggressively at the start of their disease course to prevent any initial damage, thereby reducing the likelihood of future damage. aSLE studies have also shown that prevention of any initial organ damage reduces mortality risk [39], further underlining the importance of damage prevention early in the disease.

This study does have some limitations which should be acknowledged. Despite drawing on one of the largest cSLE cohorts in the world, given the rarity of cSLE, the sample size is low compared with that for aSLE studies [11]. This impacted the numbers of patients included in certain subgroups (minimal disease activity, no CS subgroups), making it impossible to fit some of the PWP Gap time models due to the low number of damage events per group. Initially, we aimed to investigate predictors of damage in a subgroup of patients with no disease activity (AMS = 0), to assess CS-associated damage in the absence of lupus disease activity. However, there were only three patients in this subgroup, and analyses were therefore not possible. Additional variables to be analysed in the future should include aPLs/presence of APS, as aSLE studies have found this to be a predictor of damage accrual [40, 41]. Unfortunately, the current study was unable to evaluate this due to difficulty assessing whether a patient had persistent antibody levels after 12 weeks due to the manner of data collection. The UK JSLE Cohort Study collects data on whether i.v. methylprednisolone has been given since the last study visit but does not collect information on the exact dose or number of pulses. Therefore, it is not possible to provide detailed data on different i.v. methylprednisolone doses and dosing regimens, and their relation to damage in JSLE. With regards to the time-adjusted mean prednisolone dose, there may be instances where sudden increases or decreases in prednisolone may have been missed, as this method of averaging out prednisolone assumed linear tapering. This variable could, therefore, be re-evaluated in future clinical trial data to get a more accurate reflection of the effects of prednisolone on damage in JSLE. It is important to note that this study describes associations and not causation, and further studies are required to gain further insight into the associations described. Future larger international studies combining existing cohorts are warranted to validate these results and enable further exploration of predictors of damage among the smaller patient subgroups, with a longer follow-up period.

## Conclusion

In conclusion, this study has demonstrated that methylprednisolone exposure, AMS score and PGA score are all factors that are associated with damage accrual in JSLE patients. Within the low disease activity subgroup multivariable model, AMS score was not a significant predictor of damage accrual, suggesting that increases in the SLEDAI-2K score up to a maximum of 4 points, does not impact significantly on damage accrual. This supports current recommendations that JSLE patients should aim to reach and maintain at least a cLLDAS state, preferably aiming for remission utilizing a T2T approach. These data also highlight the importance of CS minimization, and the need to review current recommendations for CSs, to reduce the risk of damage without compromising lupus control.

## Supplementary Material

keae592_Supplementary_Data

## Data Availability

Data are available on reasonable request.
