# Supplementary material for: Contributors to organ damage in childhood lupus: corticosteroid use and disease activity
Source: Rheumatology (Oxford). 2024 Oct 22;64(5):3028–38. doi: 10.1093/rheumatology/keae592 (PMC12048058; doi:10.1093/rheumatology/keae592)
Supplement: keae592_Supplementary_Data [file keae592_supplementary_data.zip › keae592_Supplementary_Data/rhe-24-1528-File006.docx]

**Supplementary material**

**Supplementary Figure S1:**

**S1: Heatmap depicting the correlation between factors considered for the models**. Pearson’s correlation test results are shown and factors with a correlation of ≥ 0.5 were excluded. Only any prednisolone was excluded (correlation of 0.5 with adjusted mean prednisolone). Immunosuppressants included: azathioprine, mycophenolate mofetil, cyclosporin, methotrexate, intravenous immunoglobulin G, rituximab and cyclophosphamide. AMS: Adjusted Mean Systemic Lupus Erythematosus Disease Activity Index 2000 (SLEDAI-2K), PGA: Physicians Global Assessment, SDI: Systemic Lupus International Collaborative Clinics/American College of Rheumatology Damage Index.

**Supplementary Figure S2:**

**S2: Graphical depiction of AMS score calculation**. AMS: Adjusted Mean Systemic Lupus Erythematosus Disease Activity Index 2000 (SLEDAI-2K).

**Supplementary Figure S3:**

**S3: Venn diagram of damage experienced in the whole patient cohort.**

**Supplementary Table S1: Statistical differences between factors among the low disease activity and moderate-high disease activity subgroup**.

| **Clinical/Demographic Factors** | **Description** | **Adjusted Mean**  **SLEDAI-2K ≤4 Patients**  **(n=195, Low Disease Activity)** | **Adjusted Mean**  **SLEDAI-2K >4 Patients**  **(n=210,**  **Moderate-High Disease Activity)** | **Difference Between low disease activity and moderate-high disease activity SLEDAI-2K groups** |
| --- | --- | --- | --- | --- |
| **Median number of visits (IQR)** | Per patient | 10 (6-16) | 10 (5-15) | p=0.50~ |
| **Median time between first ever visit and diagnosis date (IQR)** | In months | 0.0 (0.0-3.3) | 0.0 (0.0-0.9) | p=0.36~ |
| **Median disease duration (IQR)** | In years | 4.1 (2.5- 6.5) | 3.6 (1.9-5.6) | **p=0.02~** |
| **Median time interval between visits (IQR)** | In months | 3.5 (2.3-5.4) | 3.0 (1.8-4.4) | **p<0.001~** |
| **Gender**  **n (%)** | Female | 162 (83.1) | 176 (83.8) | p= 0.95^ |
| **Median age (IQR)** | At diagnosis | 12.5 (10.1-13.9) | 12.9 (10.6-14.8) | **p=0.03~** |
| **Ethnicity***  **n (%)** | African/Caribbean  Asian  White | 27 (14.7)  51 (27.7)  106 (57.6) | 37 (18.8)  64 (32.5)  96 (48.7) | p=0.21^ |
| **Baseline Organ Damage, SDI Score****  **n (%)** | No Damage (0)  Mild Damage (1)  Moderate Damage (2)  Severe Damage (≥3) | 160 (86.0)  21 (11.3)  2 (1.1)  3 (1.6) | 166 (82.2)  25 (12.4)  6 (3.0)  5 (2.4) | p=0.41^ |
| **New damage accrued during follow up period, n (%)** | No Damage (0)  Mild Damage (1)  Moderate Damage (2)  Severe Damage (≥3) | 0 (0)  22 (55.0)  9 (22.5)  9 (22.5) | 0 (0)  32 (54.2)  11 (18.6)  16 (27.1) | p=0.83^ |
| **Median of ACR score at last visit (IQR)** | At baseline | 5 (4-6) | 6 (5-7) | **p<0.001~** |
| **Any steroids during follow-up**  **n (%)** | Yes | 169 (86.7) | 195 (92.9) | p=0.06^ |
| **Prednisolone during follow-up**  **n (%)** | Yes | 166 (85.1) | 192 (91.4) | p=0.07^ |
| **Time-adjusted mean prednisolone (IQR)** | During follow-up | 3.8 (1.1-7.4) | 7.6 (4.0-11.9) | **p<0.001~** |
| **Methylprednisolone during follow-up**  **n (%)** | Yes | 76 (39.0) | 119 (56.7) | **p=0.001^** |
| **Number of methylprednisolone pulses** | No pulses ever  <5 pulses ever  >=5 pulses ever | 119 (61.0)  53 (27.2)  23 (11.8) | 91 (43.3)  83 (39.5)  36 (17.1) | **p=0.002^** |
| **Hydroxychloroquine during follow-up**  **n (%)** | Yes | 185 (94.9) | 192 (91.4) | p=0.24^ |
| **Immunosuppressants during follow-up**  **n (%)** | Yes | 195 (100.0) | 208 (99.0) | p=0.51^ |
| **Baseline renal disease BILAG score**  **n (%)** | Severe-moderately active disease | 62 (31.8) | 71 (33.8) | p=0.75^ |
| **Time-adjusted mean PGA score (IQR)** | During follow-up | 0.08 (0.02-0.23) | 0.17 (0.05-0.40) | **p<0.001~** |

**ST1:** ~Mann-Whitney U test applied. ^Chi-squared test applied. *Ethnicity data was not available for 11 patients. **SDI data was not available for 20 patients. Immunosuppressants included: azathioprine, mycophenolate mofetil, cyclosporin, methotrexate, intravenous immunoglobulin G, rituximab and cyclophosphamide. AMS: Adjusted Mean Systemic Lupus Erythematosus Disease Activity Index 2000 (SLEDAI-2K), ACR: American College of Rheumatology, BILAG: British Isles Lupus Assessment Group, IQR: interquartile range, PGA: Physicians Global Assessment, SDI: Systemic Lupus International Collaborative Clinics/American College of Rheumatology Damage Index
