# Supplementary figures and images for: Contributors to organ damage in childhood lupus: corticosteroid use and disease activity
Source: Rheumatology (Oxford). 2024 Oct 22;64(5):3028–38. doi: 10.1093/rheumatology/keae592 (PMC12048058; doi:10.1093/rheumatology/keae592)

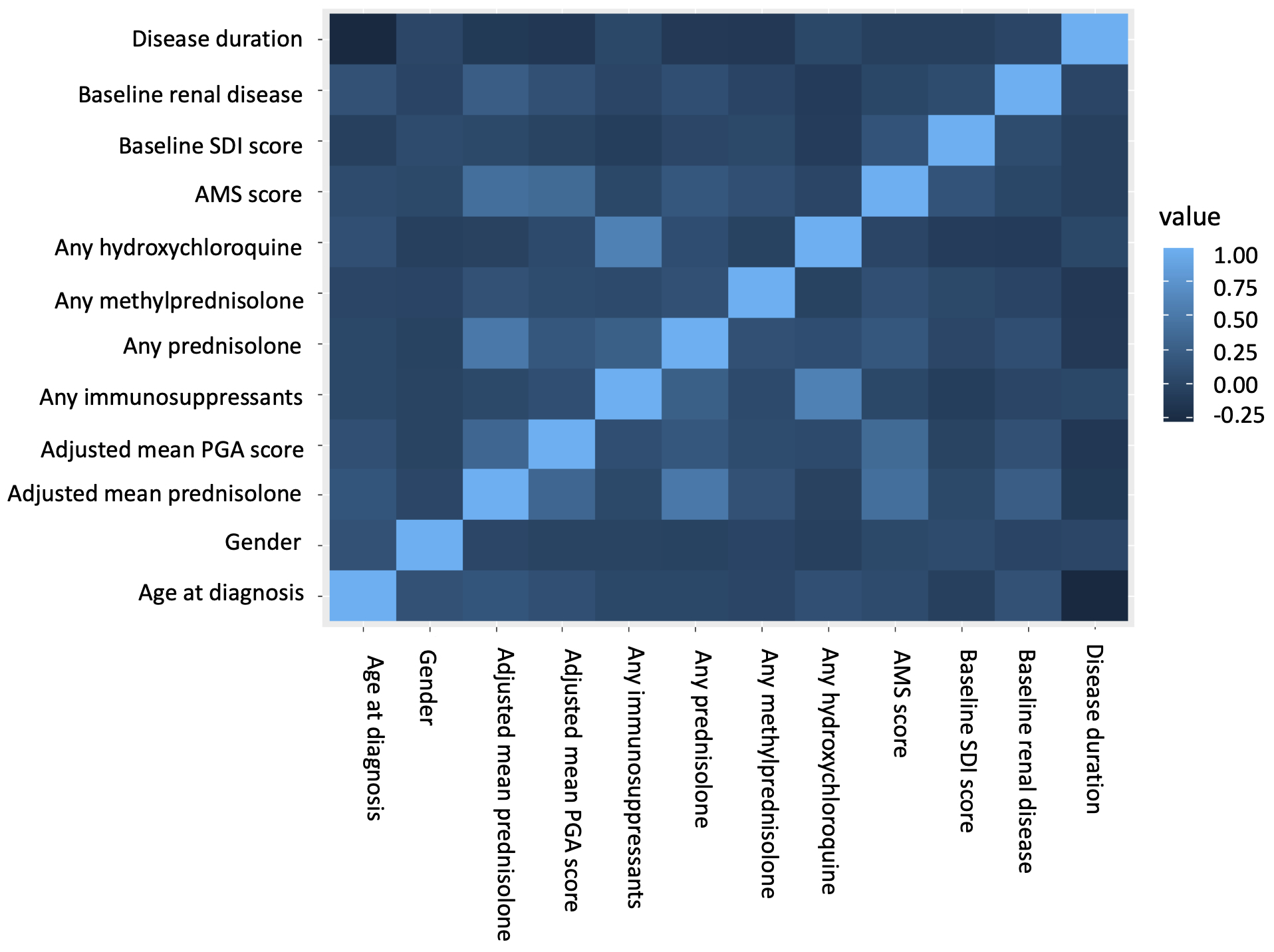

Supplement: keae592_Supplementary_Data [file keae592_supplementary_data.zip › keae592_Supplementary_Data/rhe-24-1528-File007.jpg]

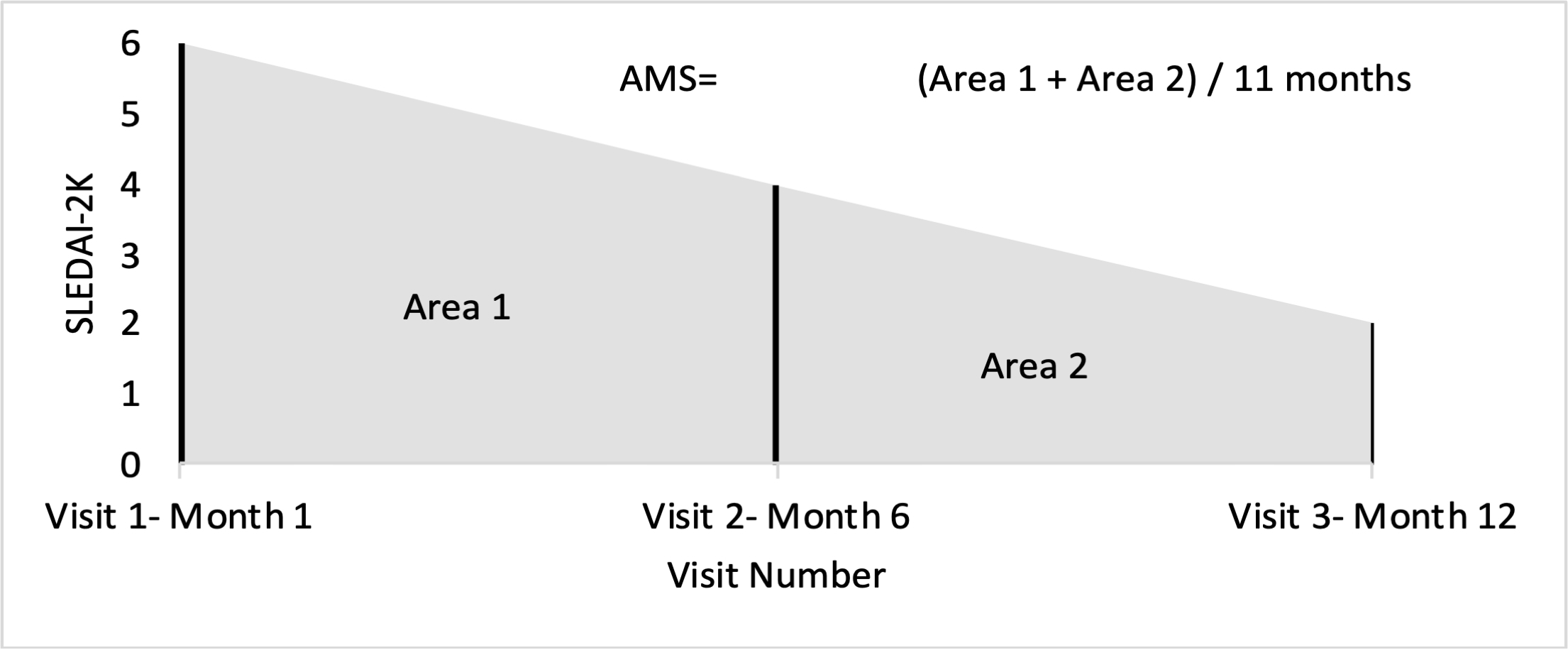

Supplement: keae592_Supplementary_Data [file keae592_supplementary_data.zip › keae592_Supplementary_Data/rhe-24-1528-File008.jpg]

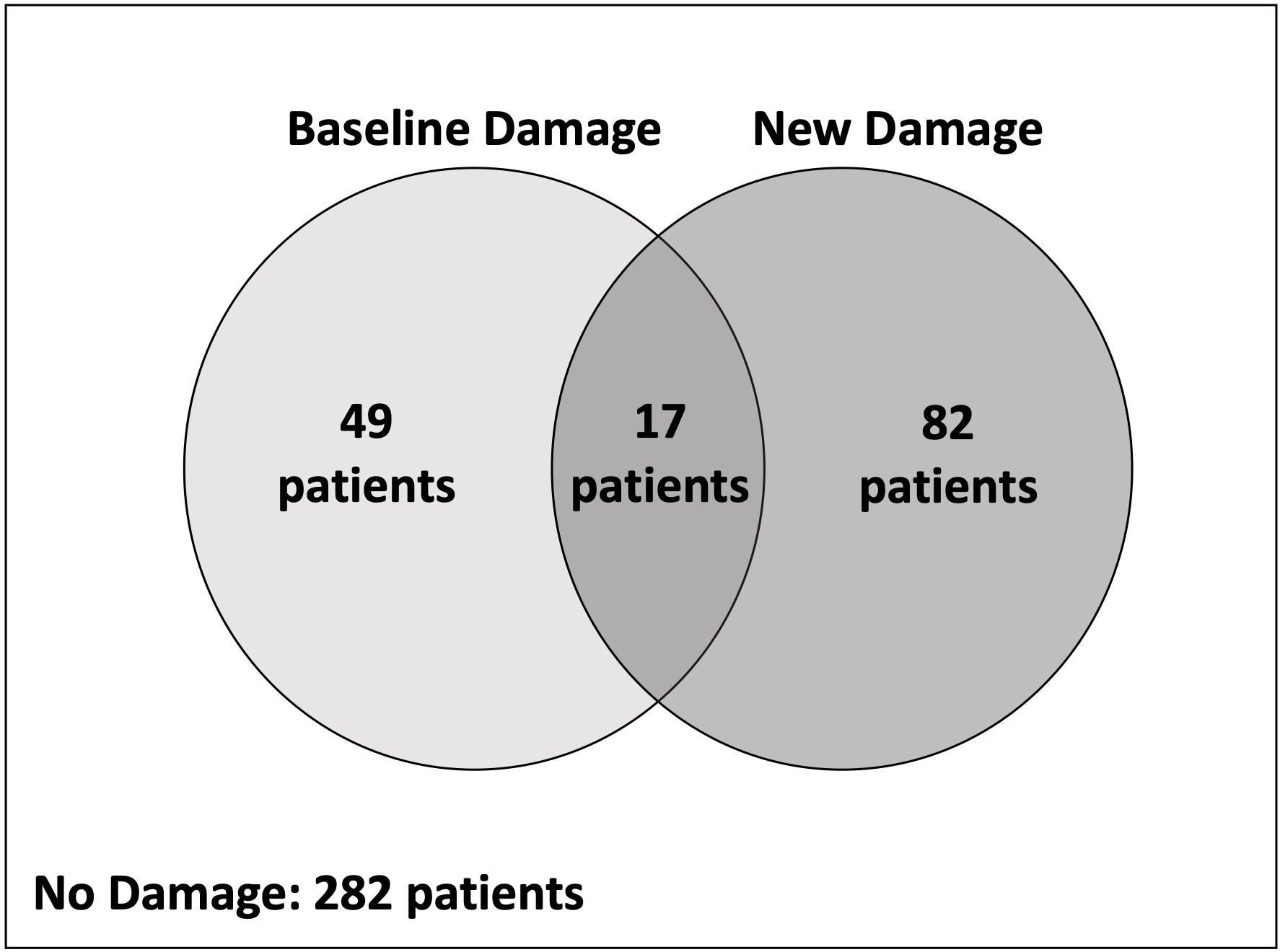

Supplement: keae592_Supplementary_Data [file keae592_supplementary_data.zip › keae592_Supplementary_Data/rhe-24-1528-File009.jpg]
